# High Order Split Operators for the Time-Dependent Wavepacket method of Triatomic Reactive Scattering in Hyperspherical Coordinates

**DOI:** 10.3390/e21100979

**Published:** 2019-10-08

**Authors:** Umair Umer, Hailin Zhao, Syed Kazim Usman, Zhigang Sun

**Affiliations:** 1 State Key Laboratory of Molecular Reaction Dynamics and Center for Theoretical and Computational Chemistry, Dalian Institute of Chemical Physics, Chinese Academy of Sciences, Dalian 116023, China; umairumer@dicp.ac.cn (U.U.); zhaohl@dicp.ac.cn (H.Z.); kazimusman@dicp.ac.cn (S.K.U.); 2 University of Chinese Academy of Sciences, Beijing 100049, China

**Keywords:** APH coordinates, scattering, splitting propagator

## Abstract

Since the introduction of a series of methods for solving the time-dependent Schrödinger equation (TDSE) in the 80s of the last centry, such as the Fourier transform, the split operator (SO), the Chebyshev polynomial propagator, and complex absorbing potential, investigation of the molecular dynamics within quantum mechanics principle have become popular. In this paper, the application of the time-dependent wave packet (TDWP) method using high-order SO propagators in hyperspherical coordinates for solving triatomic reactive scattering was investigated. The fast sine transform was applied to calculate the derivatives of the wave function of the radial degree of freedom. These high-order SO propagators are examined in different forms, i.e., TVT (Kinetic–Potential–Kinetic) and VTV (Potential–Kinetic–Potential) forms with three typical triatomic reactions, H + H ${}_{2}$ , O + O ${}_{2}$ and F + HD. A little difference has been observed among the performances of high-order SO propagators in the TVT and VTV representations in the hyperspherical coordinate. For obtaining total reaction probabilities with 1% error, some of the S class high-order SO propagators, which have symmetric forms, are more efficient than second order SO for reactions involving long lived intermediate states. High order SO propagators are very efficient for obtaining total reaction probabilities.

## 1. Introduction

Modern methods to solve the time-dependent Schrödinger equation play an important role in the description of atomic and molecular processes [1,2,3,4,5]. Especially, the time-dependent wave packet method for solving reactive scattering processes has become more and more popular due to its numerical scaling advantages. Usually, the Jacobi coordinate is applied for a reactive scattering process, since in it, the Schrödinger equation has a simple form. However, the time-dependent wave packet method using the Jacobi coordinate for a reactive scattering has two primary drawbacks [6]: First, one set Jacobi coordinate is only optimal to represent one of the arrangements. In order to extract state-to-state information, one suffers from the coordinate problem [7,8]. As far as the treatment of products with three free atoms is concerned, the Jacobi coordinates are not optimal choices. In contrast, the hyperspherical coordinate deals with all arrangement channels simultaneously and equally [6,9,10] which is capable of treating all the arrangement channels efficiently using only single propagation. Recently, Zhao et al. developed an efficient interaction-asymptotic region decomposition (IARD) method, where the adiabatically adjusting, principal axes hyperspherical (APH) coordinate presented by [9] was applied for the interaction region, but the corresponding Jacobi coordinates were applied for the asymptotic regions. The IARD method is very efficient for dealing with the state-to-state reactive scattering process using the time-dependent wave packet method [11].

In a numerical simulation of the quantum reactive scattering processes by TDWP-method, the efficiency strongly depends on the two main aspects; (i) the coordinate system and the corresponding grid representation and (ii) the time propagator to evolve the wave packet. Often, these two aspects are closely dependent and one needs to carefully design the whole numerical scheme.

Time-dependent methods are very easy to implement and they have many general applications, i.e., molecular reactive dynamics, prediction of laser atom or molecule interactions, photo-dissociation processes, ultra-cold reactions, etc. The dynamics on which we perform quantum calculations are very helpful for our understanding, through which we have accumulated a large amount of knowledge about micro-mechanisms about chemical reactions, such as the quantum bottleneck state over the transition state, chemical reaction and the geometric phase phenomenon etc.

The numerical grid methods by using the fast Fourier transform (FFT) and its mapped form, pioneered by Kosloff and his co-workers, have also proven to be very efficient and convenient for quantum molecular dynamics studies [12,13,14,15,16]. The other well known method for the solution of the molecular Schrödinger equation is the discrete variable representation (DVR) method [17,18,19,20]. Both of these two method are very effective and will be applied in the present work.

Many efficient wave packet propagation methods have been proposed in the past years, too. The most efficient and accurate propagator was the Chebyshev polynomial expansion, proposed by Tal-Ezer and Kosloff [21]. The second order SO (SSO) method [22,23] is another one of the most popular propagators. One of the interesting feature of the SSO is that it conserves the norm of the wave packet, even when a large time step is used. As a result, the propagation is exceedingly stable. The later introduced Chebyshev real wavepacket (CRWP) [24,25] is also very popular in quantum molecular dynamics field.

For reactive scattering processes, Sun et al. [26] have found that when wave packet is propagated on a flat Potential energy surface using Jacobi coordinate, the SSO appeared to be more efficient than the CRWP propagator, whereas when PES with deep potential is considered, the CRWP will give more efficient results than the SSO propagator. However, the CRWP requires a large number of iterations to obtain fully converged scattering informations for deep potentials. Recently, many groups [27,28,29,30,31,32,33,34,35,36,37,38,39,40,41,42] have investigated the application of high-order SOs for solving the Schrödinger equation. Most recently Sun et al. [43,44] draw significant numerical investigations with the high-order SOs and presented several typical tri-atomic reactive scattering calculations. The splitting integrator in either exponential VTV (potential-kinetic-potential splitting) or TVT (kinetic-potential-kinetic splitting) forms were employed in the Jacobi coordinates. They found that, generally, the high-order SO gives more efficient results in TVT form as compared to VTV version. The best high-order SOs in the Jacobi coordinate in most cases are more efficient than the SSO.

The Hamiltonian operators in the APH coordinates are very different from those in the Jacobi coordinates, thus the performances of the different high-order SOs in the APH coordinate should be very different. This motivated us to investigate how high-order SO for reactive scattering calculation works with the APH coordinates. In the literature, there are many different types of hyperspherical coordinates; for details see [45,46,47,48]. The main difference among the APH coordinate and other hyperspherical coordinates [45,46,47,48] lies in the selection of kinematic-angle and orientation of the body-fixed(BF) axes [9]. In the present work, we would only focus on the APH coordinate. The contents of the paper are further divided as follows: Section 2 contains a detailed description and derivation of the theoretical methods for the APH coordinates. Section 3 contains the numerical investigation on performance of the high-order SOs with three typical H + H ${}_{2}$ , F + HD and O + O ${}_{2}$ reactions. Finally, Section 4 concludes our present work.

## 2. Theory: Coordinates System, Hamiltonian and Split Operators

### 2.1. Theory: Mass Scaled Jacobi Coordinate and Initial WavePacket

Let A, B and C be system of three atoms with masses $m_{\alpha }$ where α = A, B, C and positions $P_{\alpha }$ w.r.t SF-axis. Total mass *M*, reduced mass μ , and the scaling factor $d_{\alpha }$ for the three atoms are defined as follow:

(1a) $$M=\sum_{\alpha =A}^{C}m_{\alpha },$$

(1b) $$\mu =\frac{m_{A}m_{B}m_{C}}{M},$$

(1c) $$d_{\alpha }=\frac{m_{\alpha }}{\mu }\left(1-\frac{m_{\alpha }}{M}\right),$$

With above three relations, we can now define the mass scaled Jacobi (MSJ) coordinates [45,46]

(2a) $$s_{\eta }=P_{\eta +2}-P_{\eta +1}$$

(2b) $$S_{\eta }=P_{\eta }-\frac{m_{\eta +1}P_{\eta +1}+m_{\eta +2}P_{\eta +2}}{m_{\eta +1}+m_{\eta +2}}$$

(2c) $$r_{\eta }=d_{\alpha }^{-1}s_{\eta }$$

(2d) $$R_{\eta }=d_{\alpha }S_{\eta }$$

where, subscripts η , η+1 and η+2 representing cyclic permutation of atoms A, B and C. $r_{\eta }$ represents the vector from particle B to C, $R_{\eta }$ position vector from center of BC to A. One advantage of the MSJ coordinates is simple orthogonal transformations among different sets are “kinematic-rotations” given by angle $\chi_{\eta +1,\eta }$

(3) $$\left(\begin{array}{c} R_{\eta +1}\\ r_{\eta +1} \end{array}\right)=U\left(\chi_{\eta +1,\eta }\right)\left(\begin{array}{c} R_{\eta }\\ r_{\eta } \end{array}\right)$$

where the transformation matrix *U* is given by

(4) $$\left(\begin{array}{c} R_{\eta +1}\\ r_{\eta +1} \end{array}\right)=\left(\begin{array}{cc} cos(\chi_{\eta +1,\eta }) & sin(\chi_{\eta +1,\eta })\\ -sin(\chi_{\eta +1,\eta }) & cos(\chi_{\eta +1,\eta }) \end{array}\right)\left(\begin{array}{c} R_{\eta }\\ r_{\eta } \end{array}\right)$$

The kinematics angles are negative obtuse angles with following properties.

(5a) $$\chi_{\eta +1,\eta }=-\chi_{\eta +1,\eta }$$

(5b) $$\chi_{\eta +1,\eta }=0$$

(5c) $$\tan\left(\chi_{\eta +1,\eta }\right)=-\frac{m_{\alpha }}{\mu }$$

(5d) $$\cos\left(\chi_{\eta +1,\eta }\right)=-\frac{\mu }{m_{\eta +2}d_{\eta }d_{\eta +1}}$$

(5e) $$\sin\left(\chi_{\eta +1,\eta }\right)=-\frac{m_{\alpha }}{\mu }$$

These equations lead to the identity

(6) $$\chi_{AB}+\chi_{BC}+\chi_{CA}=2\pi$$

The MSJ coordinates are simply known as Jacobi coordinates due to extensive use in the literature. The SF or BF-axis set can be used to describe the positions of the three atoms. The six Jacobi SF coordinates consist of two orientation angles of each Jacobi vector and magnitude.

The Hamiltonian in the MSJ coordinates for triatomic reactive scattering can be written as

(7) $$\hat{H}=-\frac{\hbar^{2}}{2\mu }\frac{1}{R_{\eta }}\frac{\partial^{2}}{\partial R_{\eta }^{2}}R_{\eta }-\frac{\hbar^{2}}{2\mu }\frac{1}{r_{\eta }}\frac{\partial^{2}}{\partial r_{\eta }^{2}}r_{\eta }+\frac{{\hat{L_{\eta }}}^{2}}{2\mu R_{\eta }^{2}}+\frac{{\hat{J_{\eta }}}^{2}}{2\mu r_{\eta }^{2}}+V,$$

where $\hat{L}$ represents orbital angular momentum operator of atom A, $\hat{J}$ is the rotational angular momentum operator of BC, $\mu_{R_{\alpha }}$ is the reduced mass between the center of mass of A and BC, and the total angular momentum is given by $J=\hat{L}+\hat{J}$ .

For Wave packet calculations, the initial wave packet (IWP) was constructed using MSJ coordinates and then transformation of IWP was done in APH coordinates as described by [6]. For aforementioned problem, the initial wave packet in SF frame $\left(v_{0},j_{0},l_{0}\right)$ can be simply constructed as a wave function expanded in the SF MSJ coordinates as under:

(8) $$\psi_{\eta v_{0}j_{0}l_{0}}^{JM\in }\left(t=0\right)=\sum_{\eta v_{0}j_{0}l_{0}}\frac{1}{R_{\eta }r_{\eta }}G\left(R_{\eta }\right)\phi_{v_{0}j_{0}}\left(r_{\eta }\right)|JMj_{0}l_{0}\in \rangle ,$$

where $|JMj_{0}l_{0}\in \rangle$ represents quantum numbers in SF-representation with the parity $\in ={\left(-1\right)}^{j_{0}+l_{0}}$ , the ro-vibrational eigenfunction of diatom BC is $\phi_{v_{0}j_{0}\left(r_{\eta }\right)}$ , and the shape of the initial wave function along the translational coordinate is Gaussian function $G(R_{\eta })$ and is given by

(9) $$G\left(R_{\eta }\right)={\left(\frac{2}{\pi \sigma^{2}}\right)}^{\frac{1}{4}}e^{\left(-{\frac{\left(R_{\eta }-R_{\eta }^{c}\right)}{\sigma }}^{2}\right)}e^{\left(-k_{c}R_{\eta }\right)}.$$

Using Equation (8) we can easily transform coordinates system from the MSJ to the APH coordinates.

### 2.2. Hyperspherical Coordinate for Triatomic Reactive Scattering

Consider the kinematics rotation:

(10) $$\left(\begin{array}{c} K_{R}\\ K_{r} \end{array}\right)=U\left(\chi_{\eta }\right)\left(\begin{array}{c} R_{\eta }\\ r_{\eta } \end{array}\right),$$

where $\chi_{\eta }$ continuous variable and its range is [0,2π] . The $\chi_{\eta }$ differ only in origin for different choices of η and are equivalent for different choices of η .

(11) $$\chi_{\eta }=\chi_{i}-\chi_{\eta_{i}},$$

where $\chi_{\eta_{i}}$ are Jacobi kinematics angles given in Equation (5a). The kinematic-angle $\chi_{\eta }$ is selected to maximize magnitude of $K_{R}$ , thus, a vector $K_{R}$ will move towards the vector $R_{\eta }$ for any atom η which left other remaining two atom. To obtain maximum value of $K_{R}$ it can be obtained from:

(12) $$\tan\left(2\chi_{\eta }\right)=\frac{2R_{\eta }.r_{\eta }}{{R_{\eta }}^{2}-{r_{\eta }}^{2}},$$

with χ ϵ [−π,π] .

Transformations from one system $BF_{\eta }$ to anothere system $BF_{K_{R}}$ usually consist of rotations $\beta_{K_{\eta }}$ about their common y-axis. This $\beta_{K_{\eta }}$ is described by the following relation:

(13a) $$\sin\left(\beta_{K_{\eta }}\right)=\frac{r_{\eta }\sin\left(\chi_{\eta }\right)\sin\left(\Theta_{\eta }\right)}{K_{R}}$$

and

(13b) $$\cos\left(\beta_{K_{\eta }}\right)=\frac{R_{\eta }\cos\left(\chi_{\eta }\right)+r_{\eta }\sin\left(\chi_{\eta }\right)\cos(\Theta_{\eta })}{K_{R}}$$

here

(13c) $$K_{R}=\sqrt{r_{\eta }^{2}{\sin(\chi_{\eta })}^{2}.{\sin(\Theta_{\eta })}^{2}+{\left(R_{\eta }\cos\left(\chi_{\eta }\right)+r_{\eta }\sin\left(\chi_{\eta }\right)\cos(\Theta_{\eta })\right)}^{2}}$$

The above system of equations are mapping between Jacobi and APH coordinates and will be used for transformation between these coordinates [9].

In the APH system, the hyperradial ρ represents the radial part, and two angular parts θ , $\chi_{i}$ are internal coordinates. The size of the triatomic system is described by hyperradial ρ and shape of the system is given by hyperangles. The angle θ is a “bending” angle, it varies the triatomic shape i.e., from an equilateral triangle θ=0 to a collinear geometry θ=π/2 , and for collinear geometry $\chi_{i}$ represents ratio of $r_{\eta }$ to $R_{\eta }$ for every fixed value ρ . These internal coordinates are defined as:

(14a) $$\rho =\sqrt{K_{r}^{2}+K_{R}^{2}}$$

(14b) $$\theta =\frac{\pi }{2}-2\tan^{-1}\left(\frac{K_{r}}{K_{R}}\right)$$

$\chi_{i}$ is defined in Equation (11). Here ρ ϵ [0,∞] and θ ϵ $\left[0,\frac{\pi }{2}\right]$ . The internal coordinates deals with all arrangement channels equally.

The relation between internal coordinates of the MSJ coordinates in terms of APH coordinates is given as:

(15a) $$R_{\eta }=\frac{\rho }{\sqrt{2}}\sqrt{(1+\sin\theta \cos2(\chi_{i}-\chi_{\eta i}))}$$

(15b) $$r_{\eta }=\frac{\rho }{\sqrt{2}}\sqrt{(1-\sin\theta \cos2(\chi_{i}-\chi_{\eta i}))}$$

and

(15c) $$\cos\Theta_{\eta }=\frac{\sin\theta \sin2(\chi_{i}-\chi_{\eta i})}{\sqrt{1-\sin^{2}\theta \cos^{2}2\left(\chi_{i}-\chi_{\eta i}\right)}}$$

and also internal coordinates of APH system in term of the MSJ are as:

(16a) $$\rho =\sqrt{R_{\eta }^{2}+r_{\eta }^{2}}$$

(16b) $$\tan\theta =\frac{\sqrt{{\left(R_{\eta }^{2}-r_{\eta }^{2}\right)}^{2}+4\left(R_{\eta }^{2}r_{\eta }^{2}\right)}}{2R_{\eta }r_{\eta }\sin\Theta_{\eta }}$$

and

(16c) $$\tan\left[2\left(\chi_{i}-\chi_{\eta_{i}}\right)\right]=\frac{2R_{\eta }.r_{\eta }}{{R_{\eta }}^{2}-{r_{\eta }}^{2}},$$

The Hamiltonian in APH coordinates is defined as:

(17) $$\begin{array}{c} H_{APH}=\frac{\hbar^{2}}{2\mu \rho^{5/2}}\frac{\partial^{2}}{\partial \rho^{2}}\rho^{5/2}-\frac{\hbar^{2}}{2\mu \rho^{2}}\left[\frac{4}{\sin2\theta }\frac{\partial }{\partial \theta }\sin2\theta \frac{\partial }{\partial \theta }+\frac{1}{\sin^{2}\theta }\frac{\partial^{2}}{\partial^{2}\chi_{i}}\right]\\ +\frac{15\hbar^{2}}{8\mu \rho^{2}}+\frac{1}{\mu \rho^{2}}\left[\frac{A_{\theta }+B_{\theta }}{2}J+\left(C_{\theta }-\frac{A_{\theta }+B_{\theta }}{2}\right)J_{z}^{2}\right]\\ +\frac{1}{2\mu \rho^{2}}\left[\frac{A_{\theta }-B_{\theta }}{2}\left(J_{+}^{2}+J_{-}^{2}\right)+\hbar D_{\theta }\left(J_{+}+J_{-}\right)\frac{\partial }{\partial \chi_{i}}\right]+V\left(\rho ,\theta ,\chi \right) \end{array}$$

where V(ρ,θ,χ) is potential energy, J represents the Total Angular Momentum,

(18) $$J_{\pm }=J_{x}\pm iJ_{y}$$

are the lowering and raising operators, and

(19) $$\begin{array}{cccccccc} A_{\theta } & =\frac{1}{1+\sin\theta }, & B_{\theta } & =\frac{1}{2\sin^{2}\theta }, & C_{\theta } & =\frac{1}{1-\sin\theta },\; & D_{\theta } & =\frac{\cos\theta }{\sin^{2}\theta } \end{array}$$

In calculations, for hyperradial “ ρ ” sine-DVR, for kinematics angle “ χ ” Fourier DVR, and finite basis representation (FBR) of spherical harmonic basis “ $y_{jK}\left(\theta_{\alpha }\right)$ ” used for angular “ θ ” coordinate.

To propagate the wavepacket in APH coordinates $\left(\rho ,\theta ,\chi_{i}\right)$ , the wavepacket can be expanded:

(20) $$\Psi^{JM\in }\left(t\right)=\sum_{K}\frac{4}{\rho^{5/2}}{\bar{D}}_{MK}^{J\in *}\left({\vec{\Omega }}_{\eta }\right)\psi^{JK\in }\left(\rho ,\theta ,\chi_{i},t_{0}\right),$$

where ${\bar{D}}_{MK}^{J\in *}\left({\vec{\Omega }}_{\eta }\right)$ represents the parity-adapted normalized rotation matrix, which depend only on Euler angles ${\vec{\Omega }}_{\eta }$ ,

(21) $${\bar{D}}_{MK}^{J\in *}\left(\vec{\Omega }\right)=\sqrt{\frac{2J+1}{8\pi^{2}\left(1+\delta_{K0}\right)}}\left[D_{KM}^{J*}\left({\vec{\Omega }}_{\eta }\right)+\in {\left(-1\right)}^{\left(J+K+\in \right)}D_{-KM}^{J*}\left({\vec{\Omega }}_{\eta }\right)\right],$$

where ∈ = ${\left(-1\right)}^{j+l}$ is the parity of the system, with *l* is the total orbital angular momentum quantum number and *K* is the projection of the total angular momentum *J* on the BF z axis and $D_{MK}^{J\epsilon }\left({\vec{\Omega }}_{\eta }\right)$ is the Wigner rotation matrix. The wave function $\psi^{JK\in }\left(\rho ,\theta ,\chi_{i},t_{o}\right)$ only depends on the internal coordinates $\left(\rho ,\theta ,\chi_{i}\right)$ and APH Hamiltonian on the *K* part of the wave-packet is:

(22) $$\begin{array}{c} H_{APH}\psi^{JK\in }\left(\rho ,\theta ,\chi_{i},t_{0}\right)=(-\frac{\hbar^{2}}{2\mu }\frac{\partial^{2}}{\partial \rho^{2}}+\frac{15\hbar^{2}}{8\mu \rho^{2}}-\frac{\hbar^{2}}{2\mu \rho^{2}}\left[\frac{4}{\sin2\theta }\frac{\partial }{\partial \theta }\sin2\theta \frac{\partial }{\partial \theta }+\frac{1}{\sin^{2}\theta }\frac{\partial^{2}}{\partial^{2}\chi_{i}}\right]\\ +V\left(\rho ,\theta ,\chi \right)+\frac{1}{\mu \rho^{2}}\left[\frac{A_{\theta }+B_{\theta }}{2}\hbar J(J+1)+\left(C_{\theta }-\frac{A_{\theta }+B_{\theta }}{2}\right)\hbar^{2}K^{2}\right]\\ +\frac{1}{2\mu \rho^{2}}\sum_{K^{\prime }=0}^{J}\langle {\hat{D}}_{KM}^{J\in *}|\frac{A_{\theta }-B_{\theta }}{2}\left(J_{+}^{2}+J_{-}^{2}\right)+\hbar D_{\theta }(J_{+}+J_{-})\frac{\partial }{\partial \chi_{i}}|{\hat{D}}_{K^{\prime }M}^{J\in *}\rangle )\psi^{JK\in }\left(\rho ,\theta ,\chi_{i},t_{0}\right) \end{array}$$

i.e., the wave function $\psi^{JK\in }\left(\rho ,\theta ,\chi_{i},t_{0}\right)$ , can be expanded as:

(23) $$\psi^{JK\in }\left(\rho ,\theta ,\chi_{i},t_{0}\right)=\sum_{n,m,j}F_{nmj}u_{n}\left(\rho \right)\phi_{m}\left(\chi \right)y_{jK_{\alpha }}\left(\theta \right),$$

here *n*, *m* represent basis-labels, u(ρ) represents basis for hyperradius coordinate, ϕ(χ) basis for kinematics angles coordinates, and $y_{jK_{\eta }}=\sqrt{\frac{\left(2j+1\right)}{4\pi }}d_{K_{\eta 0}}^{j}$ is spherical harmonics. reduced Wigner rotational matrix [49] with $K_{\eta }=0$ is $d_{K_{\eta 0}}^{j}$ . $A_{\theta }$ , $B_{\theta }$ , $C_{\theta }$ and $D_{\theta }$ all are given in the Equation (19). The matrix elements of the Equation (22) can be solved analytically [9]:

(24) $$D_{mat}=\nu_{J,K}^{+}\nu_{J,K+1}^{+}\delta_{K^{\prime },K+2}-\nu_{J,K}^{-}\nu_{J,K-1}^{-}\delta_{K^{\prime },K-1}+{\left(-1\right)}^{\left(J+K+\in \right)}\nu_{J,K}^{-}\nu_{J,K-1}^{-}\delta_{K^{\prime },2-K}$$

(25) $$\begin{array}{c} \langle {\hat{D}}_{KM}^{J\in *}|\left(J_{+}^{2}+J_{-}^{2}\right)|{\hat{D}}_{K^{\prime }M}^{J\in *}\rangle =\hbar^{2}\frac{D_{mat}}{\sqrt{(1+\delta_{K,0})\left(1-\delta_{K^{\prime },0}\right)}} \end{array}$$

and

(26) $$\langle {\hat{D}}_{KM}^{J\in *}|(J_{+}+J_{-})|{\hat{D}}_{K^{\prime }M}^{J\in *}\rangle =\frac{\nu_{J,K}^{+}\delta_{K^{\prime },K+1}-\nu_{J,K}^{-}\delta_{K^{\prime },K-1}+{\left(-1\right)}^{\left(J+K+\in \right)}\nu_{J,K}^{-}\delta_{K^{\prime },1-K}}{\sqrt{(1+\delta_{K,0})\left(1-\delta_{K^{\prime },0}\right)}}$$

where $\nu_{J,K}^{\pm }$ is given by [9]: $\nu_{J,K}^{\pm }=\sqrt{\left(J\pm K+1)(J\mp K\right)}$ . The results of these equations are asymmetric top and Coriolis coupling coefficients, respectively.

### 2.3. Split Operators

The SSO is very common nowadays in a molecular quantum dynamics calculation, For completeness, we make a brief introduction about it and its high-order forms.

#### 2.3.1. Second Order Split Operator

The TD form of one-dimensional Schrödinger equation is given by:

(27) $$i\hbar \frac{\partial }{\partial t}\Psi \left(\vec{r},t\right)=\hat{H}\Psi \left(\vec{r},t\right)=\left(\hat{T}+\hat{V}\right)\Psi \left(\vec{r},t\right),$$

where *V* is potential, which depends only on *r*. Using SSO, the solution of Equation (27) can be written as

(28) $$\Psi \left(\vec{r},t+\Delta_{t}\right)=e^{-i\hat{H}\Delta_{t}}\Psi \left(\vec{r},t\right)=S_{2}\left(\Delta_{t}\right)\Psi (\vec{r},t)+O\left(\Delta_{t}^{3}\right)$$

here

(29) $$S_{2}\left(\Delta_{t}\right)=e^{i\hat{H}\Delta_{t}}=e^{-i\frac{\Delta_{t}V}{2}}e^{-i\Delta_{t}T}e^{-i\frac{\Delta_{t}V}{2}}$$

or

(30) $$S_{2}\left(\Delta_{t}\right)=e^{i\hat{H}\Delta_{t}}=e^{-i\frac{\Delta_{t}T}{2}}e^{-i\Delta_{t}V}e^{-i\frac{\Delta_{t}T}{2}}$$

The SSO in Equation (29) is named as the VTV form and in Equation (30) is named as the TVT form, for facilitating the following discussions.

#### 2.3.2. High Order Split Operator

There are a number of ways to develop the high-order SO as reported by [28,29,30,31,32,33,34,35,36,37,38,39,40,41,42], but the simplest and most straight forward way to develop a higher order SO is, product of the lower order integrators with different time steps as presented by [50,51,52] as following

(31) $$S_{2k+2}\left(\Delta_{t}\right)={\left[S_{2k}\left(\alpha_{1}\Delta_{t}\right)\right]}^{n}{\left[S_{2k}\left(\alpha_{0}\Delta_{t}\right)\right]}^{m}{\left[S_{2k}\left(\alpha_{1}\Delta_{t}\right)\right]}^{n}$$

here $\alpha_{1}={\frac{1}{\left(2n-2nm^{2k}\right)}}^{\frac{1}{\left(2k+1\right)}}$ and $\alpha_{0}=\frac{1-2n\alpha_{1}}{m}$ are (2k+2)th order split propagators. The efficiency of integrator in Equation (31) comes at higher cost [53], and only 4th order operator have been studied in literature. An alternative way to construct the high-order integrator(s) is as

(32) $$S_{n}\left(\Delta_{t}\right)=S_{2}\left(\omega_{k}\Delta_{t}\right)...S_{2}\left(\omega_{1}\Delta_{t}\right)S_{2}\left(\omega_{0}\Delta_{t}\right)S_{2}\left(\omega_{1}\Delta_{t}\right)...S_{2}\left(\omega_{k}\Delta_{t}\right)$$

The optimization of the coefficient $\omega_{k}$ is quite complicated, but SOs in Equation (32) is generally more efficient than those in Equation (31). Another way to obtain higher order split integrator is:

(33) $$S_{n}\left(\Delta_{t}\right)=e^{-i\alpha_{k+1}V}e^{-i\beta_{k}T}e^{-i\alpha_{k}V}e^{-i\beta_{k-1}T}...e^{-i\alpha_{2}V}e^{-i\beta_{1}T}e^{-i\alpha_{1}V}$$

Using this procedure, upto 8th order splitting operator propagtors have been investigated [53,54,55,56]. Due to the flexibility of parameters in Equation (33), efficient results can be obtained by using Equation (33).

In our work, to facilitate the following discussion, the high-order SOs in Equations (32) and (33) are termed as A and S-Class/series, respectively, following the work of Sun et al. [43]. And all of the high-order SO propagators are denoted with the same names as given in the work of Sun et al. [43,44]. Numerical error function $\Delta_{t}^{s}$ is defined for comparing the efficiency of high-order SOs, where $\Delta_{t}^{s}$ is called effective time step or normalized time step as given by [43], and it is equal to time step ( $\Delta_{t}$ ) per stage:

(34) $$\Delta_{t}^{s}=\frac{\Delta_{t}}{N_{s}}$$

where $N_{s}$ indicates the number of the stages. The computational effort for each stage is the same as that for the SSO, thus clearly relates to numerical efficiency of high-order SO.

### 2.4. Split Operator in the APH Coordinate

For describing a reactive scattering in the APH coordinates using the wave-packet method, we follow our previous formalism [10,11]. The initial wavepacket was first construct in the MSJ coordinates as

(35) $$\psi_{\eta v_{0}j_{0}l_{0}}^{JM\in }\left(t=0\right)=\sum_{\eta v_{0}j_{0}l_{0}}\frac{1}{R_{\eta }r_{\eta }}G\left(R_{\eta }\right)\phi_{v_{0}j_{0}}\left(r_{\eta }\right)|JMj_{0}l_{0}\in \rangle ,$$

and then was transformed into the APH coordinates, where $|JMj_{0}l_{0}\in \rangle$ represents quantum numbers in SF-representation with the parity $\in ={\left(-1\right)}^{j_{0}+l_{0}}$ , the ro-vibrational eigenfunction of diatom BC is $\phi_{v_{0}j_{0}\left(r_{\eta }\right)}$ , and the shape of the initial wave function along the translational coordinate is Gaussian function $G(R_{\eta })$ and is given by Equation (9).

Wave packet propagation involves the implementation of sine transform on hyperradius in order to calculate the kinetic energy operators effect on the wavepacket. The interaction of an angular kinetic energy operator on wavepacket can be evaluated in the FBR using associated Legendre polynomials, and for evalution of potential energy, the DVR technique was applied [20,26].

Split operator in TVT form in APH coordinate are written as

(36) $$\begin{array}{cc} \Psi \left(\rho ,\theta ,\chi_{i},t+\Delta_{t}\right) & =S\left(\Delta_{t}\right)\Psi \left(\rho ,\theta ,\chi_{i},t\right)\\ & \equiv e^{-iT\Delta_{t}/2\hbar }e^{-i\hbar V\Delta_{t}/2\hbar }e^{-iT\Delta_{t}/2\hbar }\Psi \left(\rho ,\theta ,\chi_{i},t\right) \end{array}$$

or the VTV form as

(37) $$\begin{array}{cc} \Psi \left(\rho ,\theta ,\chi_{i},t+\Delta_{t}\right) & =S\left(\Delta_{t}\right)\Psi \left(\rho ,\theta ,\chi_{i},t\right)\\ & \equiv e^{-iV\Delta_{t}/2\hbar }e^{-i\hbar T\Delta_{t}/2\hbar }e^{-iV\Delta_{t}/2\hbar }\Psi \left(\rho ,\theta ,\chi_{i},t\right) \end{array}$$

where

(38) $$e^{-iT\Delta_{t}/2\hbar }=S_{2}^{T}\left(\Delta_{t}\right)\approx e^{-iT_{\chi }\Delta_{t}/2\hbar }e^{-iT_{\theta }\Delta_{t}/2\hbar }e^{-iT_{\rho }\Delta_{t}\hbar }e^{-iT_{\theta }\Delta_{t}/2\hbar }e^{-iT_{\chi }\Delta_{t}/2\hbar }$$

(39) $$\begin{array}{c} T_{\chi }=\frac{\hbar^{2}}{2\mu \rho^{2}}\left[\frac{1}{\sin^{2}\theta }\frac{\partial^{2}}{\partial^{2}\chi_{i}}\right]+\frac{1}{\mu \rho^{2}}\left[\frac{A_{\theta }+B_{\theta }}{2}J+\left(C_{\theta }-\frac{A_{\theta }+B_{\theta }}{2}\right)J_{z}^{2}\right]\\ +\frac{1}{2\mu \rho^{2}}\left[\frac{A_{\theta }-B_{\theta }}{2}\left(J_{+}^{2}+J_{-}^{2}\right)+\hbar D_{\theta }\left(J_{+}+J_{-}\right)\frac{\partial }{\partial \chi_{i}}\right], \end{array}$$

(40) $$\begin{array}{c} T_{\theta }=\frac{\hbar^{2}}{2\mu \rho^{2}}\left[\frac{4}{\sin2\theta }\frac{\partial }{\partial \theta }\sin2\theta \frac{\partial }{\partial \theta }\right], \end{array}$$

(41) $$\begin{array}{c} T_{\rho }=\frac{\hbar^{2}}{2\mu }\frac{\partial^{2}}{\partial \rho^{2}}+\frac{15\hbar^{2}}{8\mu \rho^{2}}. \end{array}$$

Higher order SO can be easily implemented by combining series of SSO in both VTV and TVT form. The important point is to carefully design local time step as discussed by [43] for the higher order SO. Due to the energy transformation, time step have upper limit for higher order SOs, which is defined by $\left(E_{\max}-E_{\min}\right)<\frac{2\pi }{\Delta t}$ , where $\left(E_{\max}-E_{\min}\right)$ is the maximal energy distribution of wavepacket.

Finally, we use an absorption potential of the following form [43] to avoid the wave function reaching towards the grid boundary along the ρ degree of freedom:

(42) $$D\left(R\right)=i\Delta_{t}C\left(\frac{\rho -\rho_{a}}{\rho_{b}-\rho_{a}}\right),\rho_{a}⩽\rho ⩽\rho_{b}=\rho_{\mathrm{end}},$$

$\rho_{a}$ is the starting point of the absorbing potential region so $\rho_{a}⩽\rho ⩽\rho_{b}$ and *C* defines the strength of the absorbing potential.

## 3. Results and Discussion

The numerical error was estimated from the total reaction probabilities, which was calculated by the flux formalism method as

(43) P(E)=1μIm[〈ΨfJK∈(E)|∂∂ρ|Ψf′JK∈(E)〉],

where $\Psi^{JK\in }\left(E\right)$ is given by

(44) $$\Psi^{JK\in }\left(E\right)=\frac{1}{a(E)}\int e^{\left(iEt\right)}\Psi^{JK\in }\left(t\right)dt,$$

where a(E) is determined by the initial wavepacket [8,26]. The error was defined as

(45) $$\sigma =\frac{1}{M}\sum_{k=1}^{M}\left|P\left(E\right)-P^{0}\left(E\right)\right|/P^{0}\left(E\right),$$

where *M* is the number of the collision energies $E_{k}$ and $P^{0}\left(E_{k}\right)$ was the converged results calculated with very small time step. For different reactions the value of *M* and range of energy are given in Table 1.

The Hamiltonian representation in APH coordinates is more complex than in Jacobi coordinates, hence, it requires smart execution of the SO to achieve efficiency and accuracy. In the Jacobi coordinate, the two radial kinetic energy operators can commute with each other, but in the APH coordinates, none of the kinetic energy operator can freely commute with others, which makes its efficient implementation quite difficult. To apply the high-order splitting operator in the APH coordinates for reactive scattering processes, the arrangement of the kinetic energy operators for high-order SO in both the TVT and VTV form is very important. In our strategy, the following forms are used to keep the symmetry and to avoid unnecessary calculations as in Equation (38): $T_{\chi }$ at outer most position, $T_{\theta }$ at central position and then $T_{\rho }$ at inner most position. Using this arrangement for the kinetic operators, the high-order SO in both the TVT and VTV form are implemented in the following calculations.

In order to demonstrate the efficiency of the high-order SOs in either the TVT or the VTV form for a reactive scattering process, H + H ${}_{2}$ , O + O ${}_{2}$ , and F + HD reactions are considered. The aforementioned chemical reactions possess variant dynamic characteristics. H + H ${}_{2}$ reaction is the easiest direct chemical reaction, while, with lasting resonance states and a deep potential well about 1.1 eV the O + O ${}_{2}$ chemical reaction is most complex. However, F + HD is direct but have a Fashbach resonances which makes it interesting. Therefore, these reactions gives a comprehensive view for understanding the performance of the high-order SO propagator for triatomic reactions in the APH coordinate.

**Figure 1:**
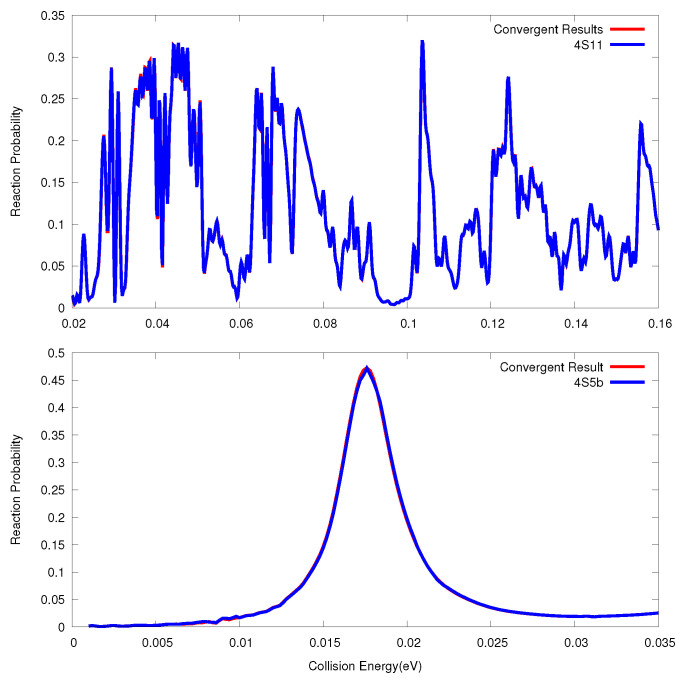
The Total reaction probabilities for the O + O ${}_{2}$ (upper) and F+HD (bottom) reactions for *J* = 0, calculated using high-order SOs using very small time step (Convergent) and using the time steps which are capable of giving results with errors of about 1%.

We plotted total reaction probabilities of the O + O ${}_{2}$ reaction and F + HD reaction, in Figure 1, calculated using high-order SOs with very small time steps and with time steps which are capable of giving results with errors of about 1%. We could see that in principle, the difference between them is very small and hard to discern by eye. It is also seen that there are many peaks in the probabilities for the O + O ${}_{2}$ reaction, which implies that there are many long lived resonance intermediate states in the potential well. There is only one peak in the probabilities for the F + HD reaction, which is the ground Feshbach resonance state in the adiabatic potential energy curve D⋯HF ( $v^{\prime }$ = 3). This state resides in the van der Waals well in the product channel. The total reaction probabilities of H + H ${}_{2}$ reaction can be found in many studies and we do not plot them here.

### 3.1. H + H ${}_{2}$ Reaction

The adiabatic BKMP2 PES [57] is employed for this reaction in our calculations to examine the performance of the high-order SOs in the APH coordinate.

**Figure 2:**
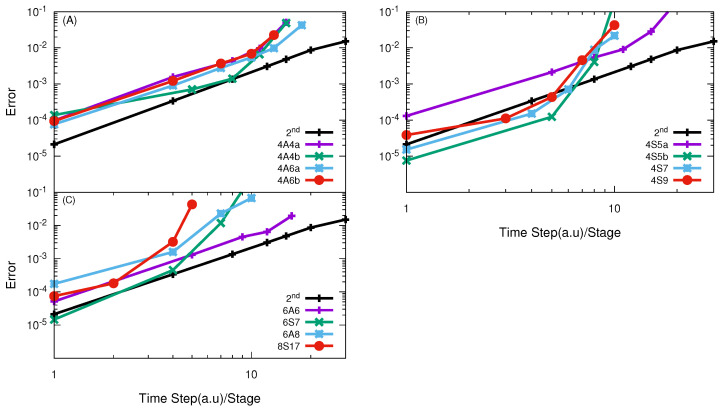
$\log_{10}$ (error) vs. $\log_{10}$ (effective time step) of different high-order SOs in the TVT form for the H + H ${}_{2}$ reaction with total angular momentum *J* = 0. In Panel (**A**) results of 4th order SO using A-class in TVT form with efficiency less than SSO propagator; panel (**B**) show the results obtained by using 4th order SO of S-class and only 4S5b converges in a high order way; Panel (**C**) represent the results of other high order split operator obtained with A and S-class and only 6S7 converges in a 2nd order way but less efficient.

In Figure 2, the numerical convergences of high-order split propagators in the APH coordinate in the TVT form (A and S class) are presented. It is seen that most of the operators are less efficient than the SSO for the whole error range, except for the S class (4S5b, 4S7) operators for results of high accuracy with small time steps. We note that the propagator named as 4S5 indicates that it is a 4th order S class propagator with four stages. Sometimes, there are more than one such propagators reported in the literature, then we further add a, b and c ... to discern.

The high-order SOs with effective time steps that are smaller than 4 or 5 a.u converge in a way of the SSO, which may suggest that the error mainly comes from the splitting of the kinetic operators. As expected, the SSO remains to be the best choice for this reaction due to the smoothness of the potential, which is similar to the conclusion in the Jacobi coordinate [43,44]. Comparison of the effective time step of the most efficient TVT form propagators in the APH coordinates and the Jacobi coordinates [43,44] at error of 1% are given in Table 2. It is interesting to see that the high-order SOs in the APH coordinate in the TVT form for this reaction are more efficient than those in the Jacobi coordinate.

**Figure 3:**
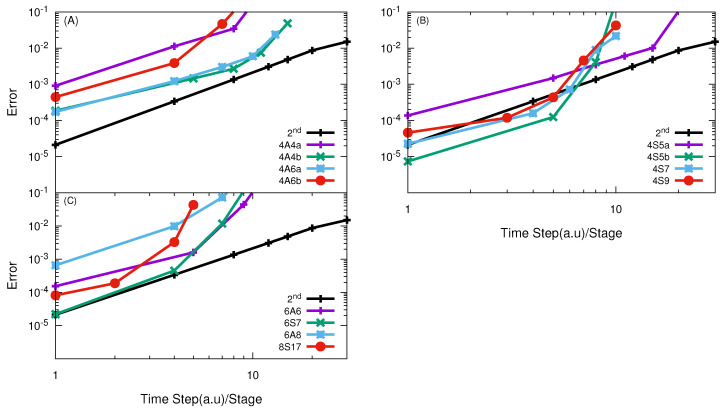
$\log_{10}$ (error) vs. $\log_{10}$ (effective time step) of different high-order SOs in the VTV form for the H + H ${}_{2}$ reaction with total angular momentum *J* = 0. In Panel (**A**) results of 4th order SO of A-class with efficiency less than SSO propagator these results are consistent with TVT form; panel (**B**) show the results obtained by using 4th order SO of S-class and again 4S5b converges in a high order way but less efficient than SSO; Panel (**C**) represent the results of other high order split operator with A and S-class and results are similar to those given in TVT form.

Similarly, in Figure 3, the results obtained using a high-order SO in the VTV form are presented. The performance of the high-order SOs in the VTV form is very similar to those in the TVT form, except for lower accuracy. There is little difference between the performance of the SSO in the TVT and the VTV form. Thus again, similar to that in the Jacobi coordinate, the SSO is the best choice among all of the SOs in the VTV form for this simple reaction.

### 3.2. O + O ${}_{2}$ Reaction

**Figure 4:**
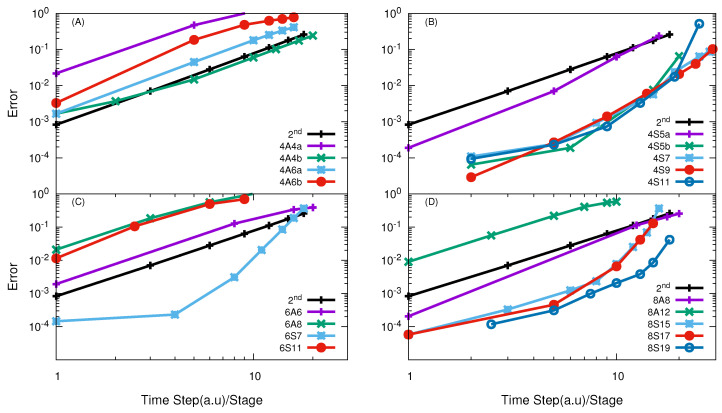
$\log_{10}$ (error) vs. $\log_{10}$ (effective time step) of different high-order SOs in the VTV form for the O + O ${}_{2}$ reaction with total angular momentum *J* = 0. In panel (**A**) results obtained using 4th order SO of A-class are given with most of these are less efficient than SSO; Panel (**B**) show the results of 4th order SO of S-class and they all converges in their higher order way and all are more efficient than SSO; Panel (**C**) show the results using 6th order SO of A and S-class, only 6S7 converges in higer order way and 2× times efficient than SSO; Panel (**D**) represent the results of 8th order SO of A and S-class, all S-class operators converges in their higher order way and more efficient than any other SO used in calculation.

For the O + O ${}_{2}$ calculations, the SSB PES [58] was applied. In Figure 4, the numerical convergences of high-order split propagators in the APH coordinate in the VTV form of both A and S class are plotted. The results in panel (A) demonstrate that the 4th-order A class propagators are not efficient for the O + O ${}_{2}$ reaction and their efficiency is lower than the SSO, even for results of high accuracy. They all converge in a way of the SSO, which suggests that the splitting is not so successful for these operators.

In comparison with these 4th-order A class propagators, the 4th-order S class propagators in VTV-form are very efficient and are able to give good results with large effective time steps, as shown in panel (B) of Figure 4. Among them, in order to obtain results with error about 1%, the 4S5b, 4S7, 4S9 and 4S11 are optimal choices to perform quantum calculations with effective time step Δt = 15.6 a.u, Δt = 16.6 a.u, Δt = 16.55 a.u and Δt = 16.96 a.u respectively. In contrast, the A class operators were proved to be most suitable choice to perform quantum calculations in the Jacobi coordinates for this reaction [43].

Panels (C) and (D) of Figure 4 presents the numerical convergence of 6th- and 8th-order propagators. Again, we see that the S class SOs are very efficient. Among them, the most efficient 6th-order method is the 6S7 and the most efficient 8th-order is the 8S19. With effective time step Δt = 11.3 a.u and 15.7 a.u, they would be able to give reaction probabilities with error less than 1%. We would like to note that, since in the current calculations for the O + O ${}_{2}$ reaction only collision energy in a very small range is considered, which allows huge total time step, the 8S19 propagator becomes effective. When collision energy in a large range is considered, the total time step becomes smaller and the 8S19 may not be the best choice anymore. From the discussion above, we see that the S class propagators in the VTV form in the APH coordinates are the optimal choices and are much more efficient than those of A class.

**Figure 5:**
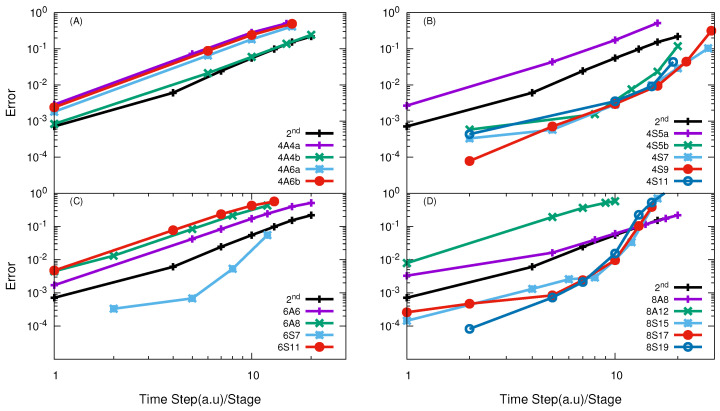
$\log_{10}$ (error) vs. $\log_{10}$ (effective time step) of different high-order SOs in the TVT form for the O + O ${}_{2}$ reaction with total angular momentum *J* = 0. In panel (**A**) results of 4th order SO of A-class are given, similar to VTV form most are these operators less efficient than SSO; Panel (**B**) show the results of 4th order SO of S-class and again they all converges in their higher order way and all are almost 3× time more efficient than SSO except 4S5a; Panel (**C**) show the results using 6th order SO of A and S-class, again similiar to VTV form only 6S7 converges in higer order way; Panel (**D**) show the results of 8th order SO of A and S-class, except A-class operator, all S-class operators converges in their higher order way and more efficient than SSO.

The numerical convergence of high-order SO in the TVT form is given in Figure 5. By comparing the results in panel (A) of Figure 5 and Figure 4, it can be seen that the 4th-order A class SOs in the TVT form exhibits a better convergence than those in the VTV form. Anyway, they are still not more efficient than the SSO.

The results in panel (B) of Figure 5 demonstrate that all the examined S class 4th-order propagators in the TVT form are more efficient than the SSO, except the 4S5a, whose efficiency is a little less than the SSO. The best one of them is 4S9, which with effective time step Δt=16.41 a.u can give results with error less than 1%.

In panels (C) and (D) of Figure 5, the numerical results of the 6th- and 8th-order SO in the TVT form are given. It is seen that the 6S7 is the most efficient one among the 6th-order SOs and the 8S19 is the best one among the 8th-order SOs. With effective time step Δt = 9.1 a.u, the 6S7 is able to give probabilities with error less than 1%. In Table 2 and Table 3 optimal propagators with the effective time step for giving error of 1% are listed for this reaction, both in the TVT and VTV form, along with the corresponding results in the Jacobi coordinates. It can be concluded that the S class propagators in the VTV form for O + O ${}_{2}$ reaction in the APH coordinates are very efficient, which is of comparable performance of the best A class SO in the Jacobi coordinate.

### 3.3. F + HD→ HF + D Reaction

**Figure 6:**
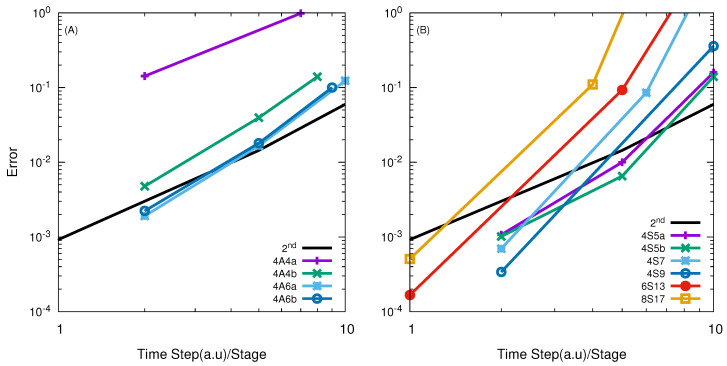
$\log_{10}$ (error) vs. $\log_{10}$ (effective time step) of different high-order SOs in the TVT form for the F + HD reaction with total angular momentum *J* = 0. Panel (**A**) represents results for the A class operators, while panel (**B**) shows results for the S class operators.

The FXZ PES [59,60] describing the F + HD reaction was used in our following calculations. Figure 6 presents the numerical convergence of high-order SO in the TVT of both A and S class. The results in panel (A) indicate that the examined 4th-order A class SO are less efficient than the SSO, and converges in a second order way. However, the results in panel (B) of Figure 6 indicate that all S class operator in TVT form shows faster convergence as a function of the effective time step. Among them, the 4S5b is the best one. With effective time step as 5.99 a.u, it is able to give reaction probabilities with errors of less than 1%. Comparison of the effective time steps of the best high-order SO in the TVT form for giving error less than 1% in the APH-coordinates and the Jacobi coordinates are presented in Table 2.

**Figure 7:**
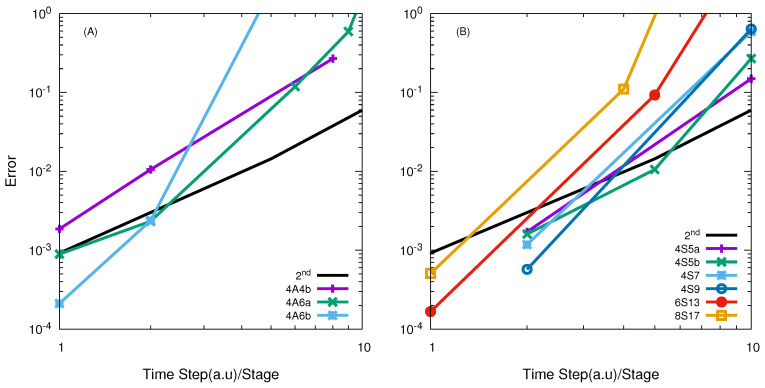
$\log_{10}$ (error) vs. $\log_{10}$ (effective time step) of different high-order SOs in the VTV form for the F + HD reaction with total angular momentum *J* = 0. Panel (**A**) represents results for the A class operators, while panel (**B**) shows results for the S class operators.

Figure 7 gives the numerical convergence for high-order A and S class SOs in the VTV form. It can be seen that these A class operators are inefficient, and their efficiency is only comparable with the SSO for very small time step per stage. They are not as good as the A class operators in the TVT form as shown in panel (A) of Figure 6.

As seen from panel (B) of Figure 7, all examined high-order S class operators in the VTV form are clearly more efficient. The 4S5b is the best one, and with effective time step $\Delta_{t}^{s}$ = 5.94 a.u, it can give reaction probabilities with errors less than 1%. Comparison of the effective time steps of the best high-order SO in the VTV form for giving error less than 1% in the APH coordinates and the Jacobi coordinates are presented in Table 3.

The 6th-order SOs only works with small time step, and similar behaviour is observed with 8th-order SOs. Numerical convergence are given in Figure 6 and Figure 7.

From the discourse above, we can conclude that the S class high-order SOs in the TVT form are a little more efficient than those in the VTV form, whereas for the A class propagators, the high-order SOs in both TVT and VTV forms have similar numerical convergence behaviours in APH coordinates for the F + HD reaction. The most important observation is that most of the examined high-order SOs for the F + HD reaction exhibit high-order numerical convergence, which is quite unusual. This fact may suggest that most high-order SOs are more efficient than the SSO for calculating the results with high accuracy for the F + HD reaction. This might result from the fact that the Feshbach resonance resides in the product channel, which is the most important region for the reaction. However, for the other two reactions, the most important region for the reaction should be the interaction region. Thus, the error comes from the kinetic operators splitting and being quite different from that in the O + O ${}_{2}$ reaction.

## 4. Conclusions

In this study, the performance of a series of high-order SOs for a triatomic reactive scattering process in the APH coordinate is examined. Since the kinetic energy operator in the APH coordinate is more complicated than the kinetic energy operator in the Jacobi coordinate, and the high-order SOs are derived using the one-dimensional model, it is not clear (a priori) if the high-order SOs are still effective. The numerical investigation suggests that the S class high-order SOs are very effective in the APH coordinate for reactions involving resonance, such as O + O ${}_{2}$ and F + HD reaction. This is different from that in the Jacobi coordinate, where the most effective high-order SOs are the ones of A class. At the same time, we notice that in the APH coordinate, the performance of the high-order SOs in the TVT and VTV form are almost the same. However, in the Jacobi coordinate, the operators in the TVT form are a little better. For the simple direct reaction H + H ${}_{2}$ , the most efficient propagator is proved to be the 2nd-order SO. This is consistent with the results given in the Jacobi coordinate.

In this work, an interesting thing is noticed for the F + HD reaction. Usually, the convergence rate of higher order SOs is of 2nd order at small time steps due to the complicated kinetic operators of the Hamiltonian in the APH coordinate, where their splitting only has a second order convergence. However, irrespective of the time step range, the high-order convergence of the SOs of the F + HD reaction is kept. This suggests that for obtaining results of high accuracy for this reaction, most of the high-order SOs are more efficient than the 2nd order SO.

Currently, there is focused interest on studying ultra-cold reactions. We can expect that the high accuracy property of high-order SOs is attractive and high-order SOs would gain more attention, where high precision results are required.

## Figures and Tables

**Table 1:** Reactions, Number of Collision Energies *M* and Range of energy. Collision energies are evenly distributed in the given range.

Reactions	M	Range of Energies
H + H ${}_{2}$	700	[0.3, 1.0]
O + O ${}_{2}$	701	[0.02, 0.16]
F + HD	341	[0.01, 0.035]

**Table 2:** Comparison of the most efficient propagators in the TVT form in the Jacobi and the APH coordinates.

	Jacobi	APH	Jacobi	APH
**Reactions**	**A Class/Time Step (a.u)**	**A Class/Time Step (a.u)**	**S Class/Time Step (a.u)**	**S Class/Time Step (a.u)**
H + H ${}_{2}$	4A4b/5.0, 4A6b/3.3	4A4b/11.9, 4A6b/13.1, 6A6/14.91	4S5b/4.5	4S5a/12.1, 4S5b/8.7
O + O ${}_{2}$	-	4A4b/5.25	-	4S9/16.41
F + HD	4A6a/9.0	4A6a/4.08	4S7, 4S9/5.1	4S5b/5.99

**Table 3:** Comparison of the most efficient propagators in the VTV form in the Jacobi and the APH coordinates.

	Jacobi	APH	Jacobi	APH
**Reactions**	**A Class/Time Step (a.u)**	**A Class/Time Step (a.u)**	**S Class/Time Step (a.u)**	**S Class/Time Step (a.u)**
O + O ${}_{2}$	4A6a/20.0	4A4b/5.05	-	4S11/16.96
F + HD	6A8/10.0	4A6a/3.41	4S7/4.5	4S5b/5.94
