# Correction: Umer et al. High Order Split Operators for the Time-Dependent Wavepacket method of Triatomic Reactive Scattering in Hyperspherical Coordinates. *Entropy* 2019, *21*, 979

**DOI:** 10.3390/e24111538

**Published:** 2022-10-26

**Authors:** Umair Umer, Hailin Zhao, Syed Kazim Usman, Zhigang Sun

**Affiliations:** 1 State Key Laboratory of Molecular Reaction Dynamics and Center for Theoretical and Computational Chemistry, Dalian Institute of Chemical Physics, Chinese Academy of Sciences, Dalian 116023, China; 2 University of Chinese Academy of Sciences, Beijing 100049, China

The authors would like to update the affiliations for Umair Umer and Syed Kazim Usman in the published publication [1]. In addition to affiliation 1, the updated affiliations should include: University of Chinese Academy of Sciences, Beijing 100049, China. The paper is prepared and supported under the University of Chinese Academy of Sciences International Student Scholarship Program.

The authors state that the scientific conclusions are unaffected. The original publication has also been updated.
